# DARC polymorphism that abrogates erythrocyte Duffy antigen expression influences MCP-1 plasma level in HTLV-1 infected individuals but are not associated with HAM/TSP

**DOI:** 10.1186/1742-4690-12-S1-P87

**Published:** 2015-08-28

**Authors:** Maria Clara FS Malta, Camila C Sales, Jacqueline C Guimarães, Poliane C Gonçalves, Daniel G Chaves, Anna Bárbara FC Proietti, Marina L Martins

**Affiliations:** 1 Fundação Hemominas, Belo Horizonte, Minas Gerais, Brazil; 2 Universidade Federal de Minas Gerais, Belo Horizonte, Minas Gerais, Brazil; 3 Interdisciplinary HTLV Research Group (GIPH

## 

Genetic host factors influence the outcome of HTLV-1 infection. Chemokines are important in the immune response against virus, and play a role in HAM/TSP pathogenesis. Duffy antigen receptor for chemokines (DARC) functions as a chemokine reservoir, and DARC polymorphisms rs12075 (A>G; FY*B>FY*A) and rs281477 (-46T>C; FY*B>FY*BES) may influence circulating concentrations of proinflammatory chemokines. We investigate whether Duffy genotypes influence HTLV-1 proviral load (pvl) level and plasma chemokines concentrations. HTLV-1 asymptomatic carries (AC=162), HAM/TSP patients (HAM=146) and seronegative individuals (SN=71) were genotyped for FY*A, FY*B and FY*BES. Quantification of plasmatic IL-8, MCP-1 and RANTES were performed by flow cytometry for all participants. HTLV-1 pvl was quantified in peripheral blood. The frequency of Duffy haplotypes was not significantly different among the three groups. Comparison of individuals with different Duffy haplotypes showed that pvl was significantly higher in HAM than in AC group, but not showed differences intragroup. IL-8 level was significantly higher in HAM than in AC and SN, and was higher in AC than in SN. Otherwise, MCP-1 was significantly higher in SN than in AC and HAM, but was not different when comparing AC and HAM. The highest RANTES level was seen in SN group, and the difference between SN and AC was significant, but not between SN and HAM, or between infected groups. Surprisingly, in a recessive genetic model, corrected by age and gender, the polymorphism -46C/C that abrogates erythrocyte Duffy antigen expression was significantly associated with lower level of MCP-1 in AC group, in HAM group and in all infected individuals, but not in SN group. We conclude that the Duffy null genotype (-46C/C) diminishes the plasma concentration of MCP-1 in HTLV-1 infected individuals, but this influence does not appear to be associated with HAM/TSP development. Financial support: Fundação Hemominas and FAPEMIG.

